# Substantial Differences between Organ and Muscle Specific Tracer Incorporation Rates in a Lactating Dairy Cow

**DOI:** 10.1371/journal.pone.0068109

**Published:** 2013-06-27

**Authors:** Nicholas A. Burd, Henrike M. Hamer, Bart Pennings, Wilbert F. Pellikaan, Joan M. G. Senden, Annemie P. Gijsen, Luc J. C. van Loon

**Affiliations:** 1 Top Institute Food and Nutrition (TIFN), Wageningen, The Netherlands; 2 Department of Human Movement Sciences, NUTRIM School for Nutrition, Toxicology and Metabolism, Maastricht University Medical Centre+ (MUMC+), Maastricht, The Netherlands; 3 Animal Nutrition Group, Wageningen University, Wageningen, The Netherlands; INSERM/UMR 1048, France

## Abstract

We aimed to produce intrinsically L-[1-^13^C]phenylalanine labeled milk and beef for subsequent use in human nutrition research. The collection of the various organ tissues after slaughter allowed for us to gain insight into the dynamics of tissue protein turnover *in vivo* in a lactating dairy cow. One lactating dairy cow received a constant infusion of L-[1-^13^C]phenylalanine (450 µmol/min) for 96 h. Plasma and milk were collected prior to, during, and after the stable isotope infusion. Twenty-four hours after cessation of the infusion the cow was slaughtered. The meat and samples of the various organ tissues (liver, heart, lung, udder, kidney, rumen, small intestine, and colon) were collected and stored. Approximately 210 kg of intrinsically labeled beef (bone and fat free) with an average L-[1-^13^C]phenylalanine enrichment of 1.8±0.1 mole percent excess (MPE) was obtained. The various organ tissues differed substantially in L-[1-^13^C]phenylalanine enrichments in the tissue protein bound pool, the highest enrichment levels were achieved in the kidney (11.7 MPE) and the lowest enrichment levels in the skeletal muscle tissue protein of the cow (between 1.5–2.4 MPE). The estimated protein synthesis rates of the various organ tissues should be regarded as underestimates, particularly for the organs with the higher turnover rates and high secretory activity, due to the lengthened (96 h) measurement period necessary for the production of the intrinsically labeled beef. Our data demonstrates that there are relatively small differences in L-[1-^13^C]phenylalanine enrichments between the various meat cuts, but substantial higher enrichment values are observed in the various organ tissues. We conclude that protein turnover rates of various organs are much higher when compared to skeletal muscle protein turnover rates in large lactating ruminants.

## Introduction

Protein intake stimulates muscle protein synthesis rates [1]. The digestion and absorption kinetics of the ingested dietary protein, and the subsequent release of amino acids in the circulation, modulates the amplitude of the stimulation of postprandial muscle protein synthesis rates [2], [3]. To accurately measure the amino acids appearance rates derived from the digestion and absorption of dietary protein requires that the labeled amino acid is incorporated directly within the dietary protein source as the absorption kinetics of free amino acids differs from dietary intact protein digestion and subsequent amino acid absorption kinetics [4]. Innovative work has demonstrated the feasibility of producing intrinsically labeled milk for the use in human nutrition research [5], [6], [7]. Our past efforts established that it is also feasible to produce intrinsically labeled meat for the assessment of protein digestion and amino acid absorption kinetics *in vivo* in humans [7]. The labeled meat provides researchers with another protein-rich food source to develop novel nutritional strategies aimed at maximizing postprandial skeletal muscle protein accretion.

Since the production of intrinsically labeled milk and meat involves a prolonged continuous intravenous infusion of L-[1-^13^C]phenylalanine in a lactating dairy cow, it provided us with an opportunity to gain information with regards to both muscle as well as organ-specific L-[1-^13^C]phenylalanine enrichments. Few studies have used stable isotope labeled amino acid administration in an attempt to assess skeletal muscle tissue and/or organ protein turnover of large livestock species, such as a dairy cow. Continuous intravenous infusions of stable isotope labeled amino acids are routinely applied to study skeletal muscle protein metabolism *in vivo* in humans [8], and some data have been collected in smaller livestock animals [9]. To the authors’ knowledge, however, no study has ever attempted to employ a constant intravenous tracer infusion protocol to allow a comprehensive assessment of muscle and various organ tissue protein enrichments. The interesting advantage of such an approach is that the constant amino acid tracer infusion allows for a steady delivery of the amino acid tracer in the blood stream. Hence, the subsequent amino acid tracer incorporation into the various tissues will provide more insight in organ and skeletal muscle tissue specific protein turnover rates.

In the current study, we continuously infused a large amount of L-[1-^13^C]phenylalanine for 96 h into a lactating dairy cow for the production of labeled milk and meat protein for subsequent use in human nutrition research. Besides collecting skeletal muscle tissue from the various skeletal muscle groups, various organs were also sampled to gain more insight in organ-specific protein turnover rates. Finally, we made comparisons of the concentrations of amino acids in proteins of the various cow meat/organ tissues against human skeletal muscle. This study was more comprehensive in nature (e.g., the quantity of meat cuts examined and the organ tissue enrichments) when compared with our previous work [7]. Naturally, applying constant stable isotope labeled amino acid infusion experiments on a large-scale basis (i.e., in numerous large livestock animals) is prohibitively expensive and, as such, our report is limited to a single lactating dairy cow. The present study provides unique insight in the dynamics of tissue turnover and provides proof of a large variety in muscle and organ specific turnover rates.

## Materials and Methods

### Animal Characteristics

One lactating Holstein-Friesian dairy cow (5^th^ parity, ∼680 kg live weight, 42 days in milk at the start of the infusion period) was selected for this experiment. The animal was fed a mixed ratio of grass silage (40.7%), maize silage (42.9%), wheat straw (3.2%), soy-rape seed mix (11.4%), lime (0.4%), salt (0.4%), and a mineral mix (0.9%) on a dry matter (DM) basis. During the experiment, the cow consumed 24.3±1.0 kg dry matter (DM) per day, which covered 84±3% of the net energy lactation (NEL) requirements and 82±3% of the protein requirements [10], [11]. The forage mixture was offered *ad libitum* 3 times daily allowing 10% orts. In addition, the cow received a commercially available concentrate (9.0 kg DM/d) divided into three equal portions of concentrate given at 6∶00, 14∶00, and 22∶00 h (provided at the milk collection times). The animal was housed in an individual tie stall and water was available *ad libitum*. The experiment and animal handling procedures were approved by the Institutional Animal Care and Use Committee of Wageningen University and carried out under the Dutch Law on Animal Experimentation.

### Stable Isotope Infusion

**Figure 1:**
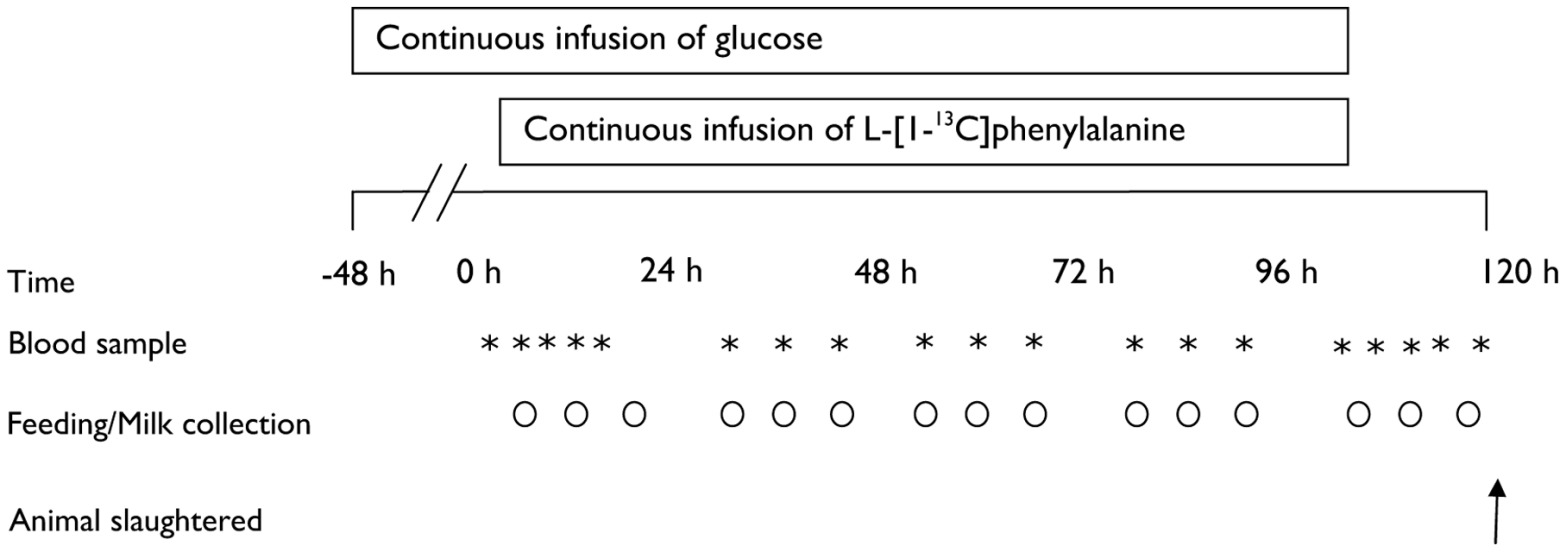
Study protocol of the cow infusion protocol.

An outline of the experimental tracer infusion protocol is shown in **Figure 1**. A total of 400 g L-[1-^13^C]phenylalanine (Cambridge Isotopes Laboratories, Andover, MA, USA) was dissolved in 40 L of an isotonic glucose (5%) solution (Braun Melsungen AG, Germany) with a final concentration of 278 mmol glucose/L and 60.5 mmol L-[1-^13^C]phenylalanine/L. The amount of tracer and the duration of the infusion was selected to achieve high labeled milk protein and labeled meat of sufficient enrichment for use in human nutrition research [7]. The infusates were stored at 4°C and allowed to warm to room temperature prior to use. Two days before the tracer infusion (−48 h), catheters (Careflow 16 gauge×300 mm with 18 gauge×70 mm needle introducer; Becton Dickinson, BD, Netherlands) were inserted percutaneously under local anaesthetics in the right and left jugular vein for the intravenous tracer infusion and blood sampling, as previously described [6]. Directly following catheterization, a glucose infusion was initiated at a rate of 116 mmol/h. The continuous glucose infusion was maintained for 48 h prior to the experimental stable isotope infusion to maximize milk protein synthesis rates [12] and reduce amino acid oxidation to maximize the tracer incorporation in endogenous protein [13]. After 48 h, the continuous infusion of glucose and L-[1-^13^C]phenylalanine was started at a rate of 7.5 mL/min (450 µmol/min) and maintained for 96 h. Milk was collected at regular intervals (6.00, 12.00, and 18.00 h of each day) during the experimental protocol. The general health of the cow was continuously monitored and all procedures were well-tolerated by the animal. Prior to the milk fractionation process, the collected milk was thawed and pooled into low and high level labeled batches. The processing and the fractionation of the milk into whey and casein protein concentrate was performed at NIZO Food Research (Ede, the Netherlands) as described in detail elsewhere [6].

### Meat and Organ Collection

The cow was transported to a commercial butchery (Henk Worst, Nijkerk, The Netherlands) and slaughtered after the infusion period. Immediately after the slaughter, large samples (200–500 g) of the heart, liver, lung, udder, kidney, rumen, small intestine, and colon were collected and stored at -18 °C until further analysis. Only a minor part of the overall tissue sample was used for the analysis (*see below*). The remaining carcass was refrigerated at 4 °C for 2 d postmortem. Afterwards, the carcass was deboned and meat cuts from the rib, shoulder and leg regions of the cow were collected. The meat cuts corresponding to portions of the chuck, foreshank, rib, short loin, sirloin, round were weighted in portions of 150 g, vacuumed sealed, and labeled before storage at −18 °C. Prior to packaging of the meat cuts, a large portion of the meat was ground into hamburger. The hamburger meat mainly originated from the brisket, plate, and flank meat cuts, but also contained other meat cut portions of the cow. The slaughter and processing of the meat followed strict Food and Consumer Product Safety Authority regulations to assure that no contamination occurred during processing of the meat and the meat products were fully qualified for human consumption.

### Cow Plasma and Protein Analysis

Plasma phenylalanine and tyrosine were derivatized to their t-butyldimethylsilyl (TBDMS) derivatives and their ^13^C enrichments were determined by electron ionization gas chromatography-mass spectrometry (GC-MS, Agilent 6890N GC/5973N MSD Little Falls, USA) using selected ion monitoring of masses 336 and 337 for unlabeled and labeled L-[1-^13^C]phenylalanine, respectively; and masses 466, 467, for unlabeled and labeled L-[1-^13^C]tyrosine, respectively.

The tissue protein-bound L-[1-^13^C]phenylalanine enrichments were determined from ∼50 mg of wet weight meat and organ tissue. The larger pieces of skeletal muscle and organ tissue samples (200–500 g) were cut. Subsequently, smaller pieces of samples (∼50 mg) were extracted from the various regions of the larger tissue sample to provide a general enrichment value. The ∼50 mg wet wt. tissue was freeze-dried, collagen, blood, and other non-relevant material were removed from the muscle or organ tissue under a light microscope. The isolated tissue mass (8 mg dry weight) was weighed and 8 volumes (8×dry weight of isolated tissue×wet/dry ratio) ice-cold 2% perchloric acid (PCA) were added. The tissue was then homogenized and centrifuged. The mixed tissue protein pellet was washed with 1.5 mL of 2% PCA and the pellet was lyophilized. Amino acids were liberated by adding 6 м HCl after which the hydrolyzed protein fraction was dried under a nitrogen stream while being heated to 120°C. The protein fraction was than dissolved in a 25% acetic acid solution and passed over a Dowex exchange resin. The amino acids were eluted with 2 м NH_4_OH, dried, and the purified amino acids were derivatized into their N(O,S)-ethoxycarbonyl ethyl esters to determine the ^13^C enrichment of tissue protein phenylalanine using gas chromatography-combustion-isotope ratio mass spectrometry (GC-C-IRMS; MAT 252, Finnigan, Breman, Germany). Standard regression curves were applied from a series of known standard enrichment values against the measured values to assess the linearity of the mass spectrometer (r^2^ = 0.99; y = 0.9894x+0.0) and to account for any isotope fractionation which may have occurred during the analysis.

### Amino Acid Concentrations

The quantification of amino acids in biological samples has been described in detail elsewhere [14]. The human skeletal muscle tissue was collected from healthy older men (n = 8) in the postabsorptive-state. The subjects were part of a larger ongoing investigation being conducted in our laboratory. The human study was approved by the Medical Ethics Committee of the Maastricht University Medical Centre, Maastricht, the Netherlands. Briefly, ∼10–15 mg of human skeletal muscle and cow meat/organ tissue were lyophilized after which they were put in a hydrolysis container containing 10 mL 6 м HCl [14]. The container was placed in an oven for 6 h at 150 C. The samples were re-dissolved in 250 µL of water, transferred to a 2 mL crimp cap vial, and lyophilized again. Subsequently, the protein hydrolysates were derivatized, and the protein amino acid concentrations were determined by HPLC as described previously [14].

### Calculations

Estimates of cow skeletal muscle and organ tissue protein fractional synthetic rates (FSR) were calculated as follows: FSR (%h^−1^) = ΔEp_/_[E_precursor_×*t*]×100. Where ΔEp is the change in protein bound L-[1-^13^C]phenylalanine enrichments in the skeletal muscle and organ tissues. E_precursor_ is the integral of the plasma free L-[1-^13^C]phenylalanine enrichment over time curve, and *t* indicates the tracer incorporation time (0–120 h).

### Statistical Analysis

Linear regression analyses were performed on the cow plasma enrichments to assess the existence of any deviation in tracer enrichment during the tracer infusion protocol.

## Results

### Plasma Enrichments

**Figure 2:**
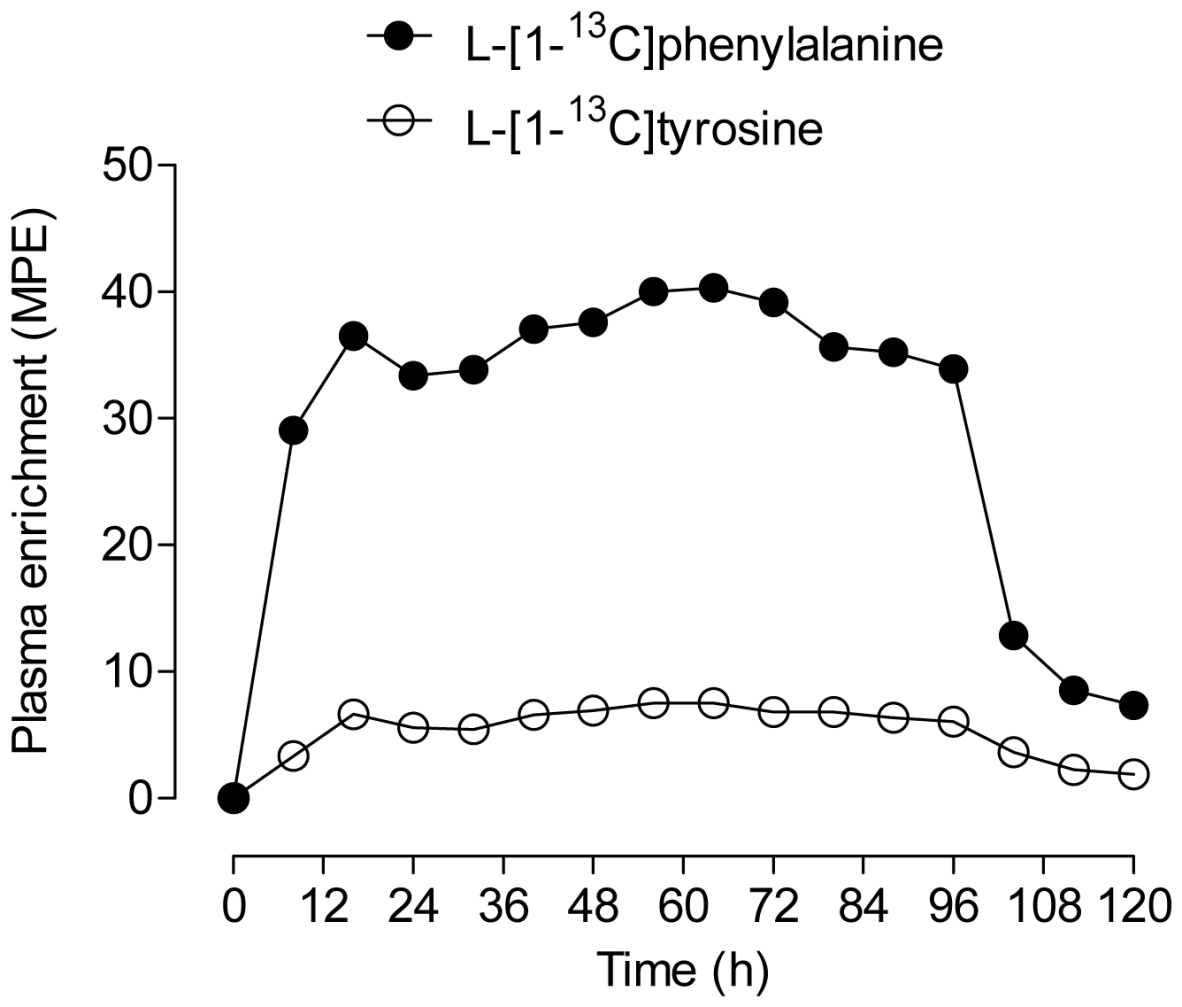
Cow plasma enrichments of L-[1-^13^C]phenylalanine and L-[1-^13^C]tyrosine before, during, and after the infusion protocol. Values are expressed as mole percent excess (MPE).

As shown in **Figure 2**, plasma free L-[1-^13^C]phenylalanine enrichments increased during the infusion. During the infusion period, the average L-[1-^13^C]phenylalanine enrichments were 36.0±1 MPE. Linear regression analysis indicated that the slope of the plasma L-[1-^13^C]phenylalanine enrichments were not significantly different from zero during the continuous infusion (P = 0.11), indicating that an isotopic steady state was achieved. In addition, plasma L-[1-^13^C]tyrosine increased to 6.2±0.3 MPE as a result of the L-[1-^13^C]phenylalanine infusion and subsequent conversion into tyrosine.

### Protein Enrichments and Tissue Synthesis Rates

**Figure 3:**
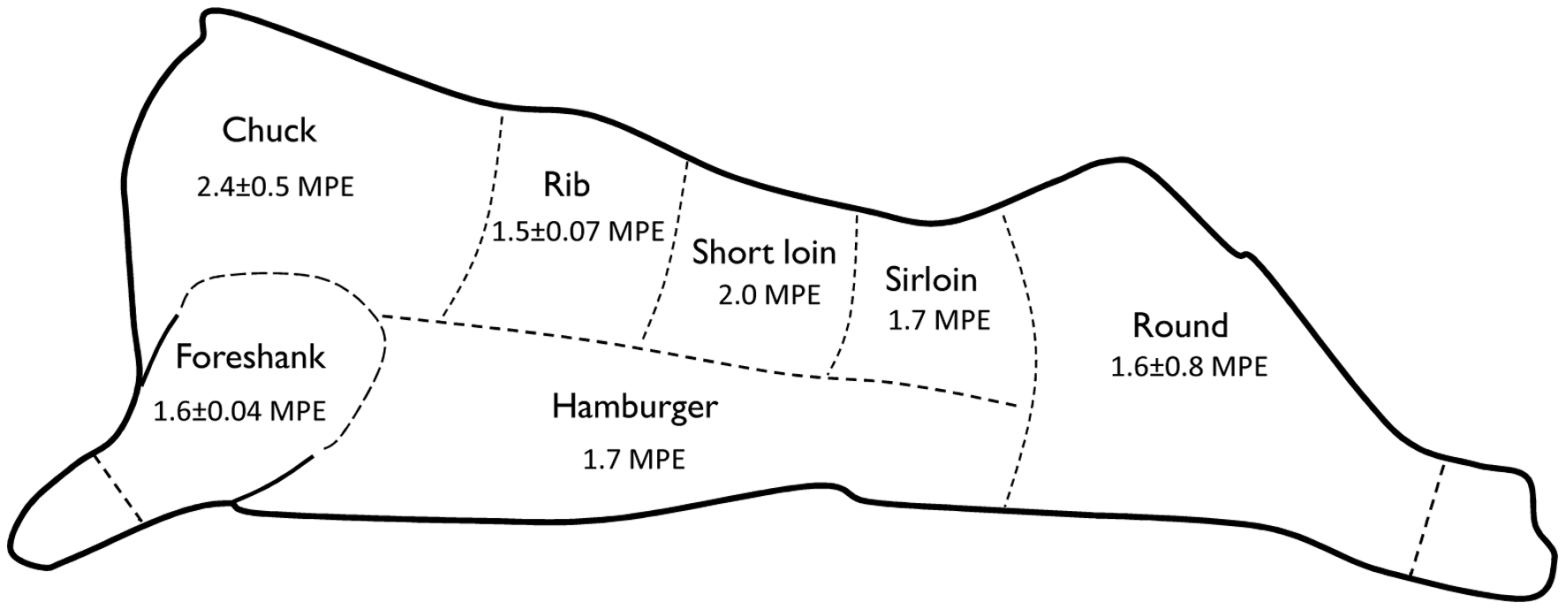
The L-[1-^13^C]phenylalanine enrichments of the meat cuts collected from different locations of a lactating dairy cow. The filet mignon is a steak cut that is, generally, positioned in the short loin steak cut. Values are expressed as mole percent excess (MPE).

**Figure 4:**
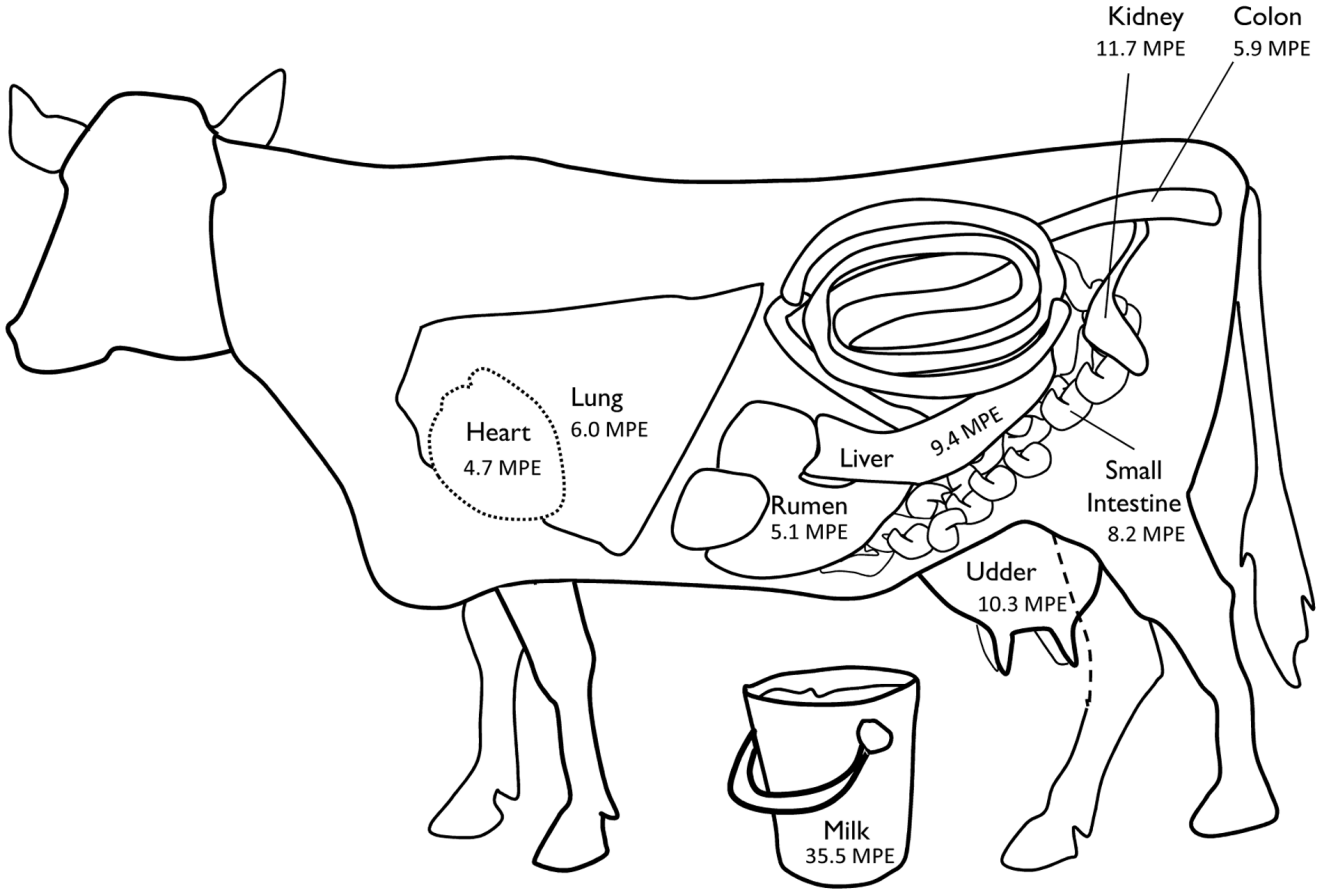
The L-[1-^13^C]phenylalanine enrichments of various collected tissues of a lactating dairy cow. The skeletal muscle tissue enrichments are a collective sum of the various collected skeletal muscle tissue. Values are expressed as mole percent excess (MPE).

The mean L-[1-^13^C]phenylalanine enrichments of the meat cuts were 1.76±0.1 MPE. The highest observed enrichment of the meat cuts was gained into the chuck steak (**Figure 3**). Notable, is the filet mignon (tenderloin) steak cut also accrued a relative high enrichment (2.0 MPE). The filet mignon is a steak cut, generally located in the short loin, which originates from the narrow end of the tenderloin (situated in the short loin/sirloin region). Of the 400 g of L-[1-^13^C]phenylalanine infused into the cow, approximately 8.4 g was recovered in the meat. Based on the estimated net meat weight of 210 kg (absent of bone and fat) and a total phenylalanine content of 8.4 kg, the recovery rate of the tracer in the meat was calculated at ∼6%. The organ tissue enrichments are presented in **Figure 4**. The various organ tissues differed in their observed L-[1-^13^C]phenylalanine enrichments which emphasizes the large variance in tissue protein turnover rates. The highest enrichments were achieved in the kidney (11.7 MPE), udder (10.3 MPE), and the liver (9.4 MPE), whereas L-[1-^13^C]phenylalanine enrichments were lower in cardiac (4.7 MPE) and skeletal muscle tissue of the cow. Using estimates of the various organ weights of a cow, the % tracer recovery rate into total organ tissue was ∼9.7%.

Estimations of the skeletal muscle and organ tissue protein synthesis rates, using the plasma free L-[1-^13^C]phenylalanine enrichment as the precursor pool, are shown in **Table 1**. The milk L-[1-^13^C]phenylalanine enrichments averaged 35.5±1.2 MPE and 7.2±3.1 MPE in the high- and low-labeled batches, respectively. Both high- and low- labeled batches were fractionated into a high- and low-labeled casein protein concentrates (28 and 23 kg, respectively) and high- and low-labeled whey protein concentrates (12 and 10 kg, respectively). The total amount of intrinsically L-[1-^13^C]phenylalanine high and low labeled casein were 4 and 3 kg, respectively. The total amount of intrinsically L-[1-^13^C]phenylalanine high and low labeled whey was 0.8 and 0.5 kg, respectively. Of the 400 g of L-[1-^13^C]phenylalanine that was infused into the cow, approximately 95 g was recovered in the collected milk. Thus, the recovery rate of the tracer in the milk was calculated to be 24%.

**Table 1:** Estimates of fractional synthesis rates (FSR) of tissue proteins and the fold difference from skeletal muscle tissue in a lactating dairy cow.

Tissue	FSR (%•h^-1^)	Fold difference from skeletal muscle tissue
Skeletal muscle	0.06	
Liver	0.31	5.3
Heart	0.15	2.6
Lung	0.20	3.4
Udder	0.34	5.8
Kidney	0.38	6.6
Rumen	0.17	2.9
Small intestine	0.27	4.6
Colon	0.19	3.3

### Amino Acid Concentrations

The human skeletal muscle, cow beef, whey and casein protein amino acid concentrations are shown in **Table 2**. Human and cow skeletal muscle tissues were similar in leucine and phenylalanine by total amino acid content (∼9% and 4%, respectively). Whey protein contained the highest leucine by total amino acid content (∼14%). The amino acid concentrations of the sampled organ tissues are shown in **Table 3**. Across the various sampled organ tissues, the bound phenylalanine by total amino acid content ranged from ∼5% (liver and kidney) to ∼3% (rumen). The total essential amino acid concentrations were similar across all the various sampled organ tissues, but were lower in total content of essential amino acids when compared with the beef samples.

**Table 2:** Protein-bound amino acid concentrations in human skeletal muscle (*n* = 10) and in cow beef, whey and casein protein (*n* = 1).

	HumanMuscle	Beef	WheyProtein	CaseinProtein
*nmol/mg of protein*				
Alanine	466±18	480	428	274
Arginine	252±7	282	127	181
Aspartic acid	498±15	511	619	422
Glutamine	889±22	929	909	1386
Glycine	409±12	464	200	205
Histidine	181±09	178	156	162
Isoleucine	255±10	301	375	354
Leucine	483±16	471	751	587
Lysine	449±12	477	585	464
Methionine	113±02	131	127	157
Phenylalanine	231±09	215	217	288
Serine	215±07	212	237	324
Threonine	245±07	253	283	251
Tyrosine	112±05	113	142	190
Valine	365±15	337	386	494
∑ EAA	2323±64	2363	2882	2757
% Leucine	9	9	14	10

EAA are the sum of His, Ile, Val, Phe, Lys, His, Met, and Thr; note that Trp, Pro, Cys were not measured. Human muscle was collected from healthy older men (n = 8). Human muscle values ± SEM.

**Table 3:** Protein-bound amino acid concentrations in the cow organ tissues (*n* = 1).

	Skeletal muscle	Liver	Heart	Lung	Udder	Kidney	Rumen	Small intestine	Colon
*nmol/mg of protein*									
Alanine	480	304	352	439	246	337	447	512	432
Arginine	282	168	196	207	137	206	273	310	284
Aspartic acid	511	304	309	337	205	333	376	432	464
Glutamine	929	496	590	569	384	541	738	744	806
Glycine	464	344	296	488	280	387	514	758	460
Histidine	178	104	98	110	58	104	82	95	97
Isoleucine	301	192	193	175	128	213	216	209	212
Leucine	471	320	317	337	212	357	330	353	374
Lysine	477	240	296	272	191	276	291	339	324
Methionine	131	64	80	69	44	81	99	91	94
Phenylalanine	215	176	151	159	94	188	149	176	165
Serine	212	180	158	195	121	190	209	217	223
Threonine	253	168	171	187	116	186	199	215	227
Tyrosine	113	48	60	57	27	68	74	178	169
Valine	337	268	256	333	183	299	294	287	309
∑ EAA	2363	1532	1563	1642	1026	1704	1660	1764	1802
% Leucine	9	9	9	9	9	9	8	7	8

EAA are the sum of His, Ile, Val, Phe, Lys, His, Met, and Thr; note that Trp, Pro, Cys were not measured.

## Discussion

The current work demonstrates the feasibility of producing intrinsically-labeled meat protein for use in human nutrition research. We demonstrate that the enrichment of organs and skeletal muscle tissue vary in a lactating dairy cow after a 96 h constant infusion of L-[1-^13^C]phenylalanine. The highest level of L-[1-^13^C]phenylalanine enrichment was observed in the kidney, udder, and liver (**Figure 3**). Skeletal muscle tissue had a lower amount of L-[1-^13^C]phenylalanine enrichment (∼4-fold) when compared with most of the organ tissues, but it has the most fundamental role in amino acid accretion due to its overall mass [15].

Protein turnover is cyclical in nature, with the synthesis of proteins being counterbalanced by their degradation rates such that tissue protein mass remains constant in a mature dry (non-lactating) well-nourished animal. It has long been recognized that fluctuations in tissue protein synthesis rates (also protein mass) can occur during a transition from dry to lactating-states in smaller sized ruminants [16], [17] and/or the nutritional-state of the animal [18], [19], [20]. Tissue protein fractional synthesis rates (FSR) are calculated by the change in tissue protein enrichment over time with respect to the precursor pool (usually plasma or tissue free) from which the labeled amino acid is incorporated into the protein [8]. Tissue protein FSR relies fundamentally on a number of basic assumptions, many of which were not adhered to within our infusion protocol [8]. Our infusion protocol was selected to achieve the highest possible L-[1-^13^C]phenylalanine enrichment in the meat/milk, and not purposely designed for the determination of muscle and organ tissue specific protein synthesis rates. We were successful in achieving a ‘steady-state’ of labeling of tracer amino acid in the plasma free amino acid pool during the infusion (**Figure 2**), and the prolonged (96 h) continuous infusion would have inevitability resulted in a steady labeling of the tissue free amino acid pool [21], an effect that would occur regardless of tissue protein turnover rates. Thus, the level of L-[1-^13^C]phenylalanine enrichment in the various collected muscle and organ tissue samples provides a proxy measurement for tissue protein turnover rates.

Cows have an innate ability to convert the relatively low quality plant-derived protein in consumed in their diet into higher quality meat and milk proteins; albeit inefficiently [22]. Here, we observed an average L-[1-^13^C]phenylalanine enrichment of 1.7±0.1 MPE in the various meat cuts, and equates to an average FSR value of ∼1.39%•d^-1^ (**Figure 3**). This observation is similar to our previous work where we produced meat that was intrinsically L-[1-^13^C]phenylalanine labeled to ∼1.4 MPE [7]. Perhaps most interesting is that L-[1-^13^C]phenylalanine enrichments were, generally, uniform over the whole skeletal muscle mass of the cow, but the enrichments were slightly higher in the meat collected from the shoulder area. Such a finding may, at least partly, relate to the increased activity of the shoulder muscles of the cow that occurred during the infusion period, as increased physical activity stimulates skeletal muscle protein synthesis rates [23]. Skeletal muscle activity increases considerably during eating and rumination in a cow [24] and the muscle free precursor pool for protein synthesis does not fluctuate greatly in various muscles of large ruminates [25], and provides support for this observation. Regional differences in fiber type composition, capillary density, and blood flow may also contribute to the variance in the proportion L-[1-^13^C]phenylalanine that was incorporated into the various meat cuts. Notable is that the enrichment in the meat, regardless of the cut, is much lower than the enrichment that is attained in the (high-labeled) milk [6], [7], and precludes its use in experiments where the metabolic fate of the ingested meat derived amino acids is directly determined in skeletal muscle tissue of the consumer [2], [3], [23], [26]. Nonetheless, the ingestion of 158 g of intrinsically L-[1-^13^C]phenylalanine labeled minced foreshank (30 g protein; 1.7 MPE) resulted in plasma L-[1-^13^C]phenylalanine enrichments between 0.3–0.7 MPE at 30–90 min in healthy young men (Burd NA, *unpublished observation*); an enrichment value that can be measured in the plasma using GC-MS analysis. The combination of continuous intravenous L-[*ring*-^2^H_5_]phenylalanine infusions with the ingestion of labeled meat provides a useful tool for the *in vivo* assessment of digestion and absorption rates following beef ingestion in humans [7].

As illustrated in **Figure 4**, there were considerable differences in L-[1-^13^C]phenylalanine enrichments in the sampled organ tissues of the lactating dairy cow. The highest observed protein bound L-[1-^13^C]phenylalanine enrichments were observed in the udder and kidney. Notable, is that changes in the mammary gland occur during lactation such that this tissue becomes more energetically expensive, metabolically active, and gains tissue protein mass [9], [27]. Mammary tissue protein synthesis rates can increase ∼15 fold in lactating (as compared to dry) goats [16]. Given this, it is clear that the higher L-[1-^13^C]phenylalanine enrichment in the udder is reflective of the increased protein synthesis rates occurring in this tissue during lactation in the dairy cow. Baracos *et al.* [17] demonstrated that kidney protein synthesis rates increased in ruminants during lactation, and can account for ∼9% (despite their small protein mass by weight) of whole body protein synthesis rates in humans [28]. Also, early work demonstrated that ∼70–90% of kidney proteins are replaced after 50 days in well fed rodents [29]. Here, we provide *in vivo* evidence that kidney protein turnover rates are high in a lactating dairy cow, as noted by a L-[1-^13^C]phenylalanine enrichment of 11.7 MPE (the highest observed enrichment amount of all the sampled tissues). The liver is classically recognized as an organ with high protein synthesis rates across various mammalian species [17], [30], [31], [32], and our data broadly agrees with this notion. Indeed, the amplitude of our reported L-[1-^13^C]phenylalanine enrichment in the liver (9.4 MPE) may be an underestimate of the ‘exact’ enrichment value (*see below*).

The gastro-intestinal tract is an area of increased metabolic activity, especially in ruminants [33], [34]. Besides data in goats and sheep [33], a paucity of information is available describing tissue protein turnover rates of larger ruminants. We collected tissue samples from the ruminal, small intestine, and colon tissues of the cow to provide insight into their protein turnover rates. We observed higher L-[1-^13^C]phenylalanine enrichments in the small intestine than the rumen or colon, which is a finding comparable to data in smaller ruminants [33]. Our *in vivo* measurements of cardiac muscle protein L-[1-^13^C]phenylalanine enrichments (4.7 MPE) demonstrated that protein turnover in the heart is dynamic as well and not as inert as often thought. A strong point of our work is that, while limited on overall number of animals studied, it provides the L-[1-^13^C]phenylalanine enrichments in multiple organ and skeletal muscle tissues within the same physiological and experimental environment (the animal served as its own control) to provide *in vivo* information on the dynamics of protein metabolism. As such, we calculated turnover rates of various tissues (see **Table 1**).

In this study, we collected the samples of the various organ and skeletal muscle tissues immediately following the butchering of the cow. We sought to collect large portions of the tissues to serve as representative pieces for the overall organ protein composition. We did not specifically seek to collect isolated portions of the organ, for example, the heart (atria *vs.* ventricles), kidney (medulla *vs.* cortex) etc. Future work is warranted to address whether there are large regional differences in protein turnover rates in various organs. In addition to this, the recycling of the amino acid label into the target tissue can be a concern during prolonged periods of a stable isotope amino acid infusion [35]. It is possible the labeled phenylalanine was incorporated into the tissue protein and subsequently released from the protein over the 96 h of tracer infusion. Since we cannot separate whether there was any re-entry of L-[1-^13^C]phenylalanine into the target tissue from protein breakdown, there was likely some ‘error’ that was introduced into the tissue protein enrichments. The extent of tracer recycling will also be dependent on the turnover rates of the sampled tissue. For slowly turning over muscle proteins, it has been estimated that ∼15% of the tracer incorporated into the muscle protein may reappear as a result of protein breakdown during a 5 day continuous stable isotope amino acid infusion in humans [36]. For organs (such as the liver) that export proteins into the blood and contain small intracellular free pools, but with high turnover rates [25], it is possible that we are providing an undervalue of the ‘true’ L-[1-^13^C]phenylalanine enrichment. Of course, the intravenous injection of a large amount of tracer and tracee amino acids (the flooding dose method) has been used to rapidly increase the labeling of all precursor pools, thereby shortening the measurement time, and presumably eliminate the uncertainty with the amount of tracer recycling occurring in a variety of organs [37]. However, the use of the large-flood dose method would not be practical to maximize the production of intrinsically labeled meat/milk for use in subsequent human nutritional studies. The presented data are simply being used to provide a general overview and lend some insight into the dynamic nature of protein turnover in various organs, most of which show much more rapid turnover when compared with skeletal muscle tissue. The fact that there might be more tracer recycling in these organ tissues only further underlines our message.

It is important to outline further that the measurement of the transfer RNA (tRNA) charged with the amino acid tracer, the true precursor for protein synthesis, is practically challenging due to the low concentrations and high turnover rates of aminoacyl-tRNA in tissue as well as the increased risk for contamination from other amino acids during its isolation/purification [38]. Workers have used other precursor pools, which are more easily accessible, as a surrogate for the aminoacyl-tRNA enrichment. The assumption is that the various free amino acid pools enrichments (i.e., extracellular plasma free or the intracellular free) closely correspond to the aminoacyl-tRNA enrichment [39]. Baumann *et al.* [39] demonstrated that the intracellular phenylalanine precursor pool may better represent the tRNA pool in the liver during a continuous stable isotope amino acid infusion, while the extracellular free pool may have greater predictive potential for the labeled tracer acylated to the tRNA in skeletal muscle and heart tissue. Notwithstanding, the aminoacyl-tRNA enrichment value is generally considered to fall in between the plasma free and intracellular free enrichment values amongst the various tissues [8]. The calculation of tissue protein FSR based on the plasma free or intracellular free precursor enrichment will provide estimates for the lower and upper limits of FSR, respectively.

Here, we used the labeling of the plasma free enrichment to represent the precursor pool for all tissues. The level error associated with any given FSR calculation using the plasma free as the precursor pool enrichment value may be more reliable for some tissues (skeletal muscle or heart) when compared to other organ tissues (liver) [39]. The estimated FSR values (**Table 1** **.**) should be considered underestimates of the true organ protein fractional synthesis rates. Data in rodents and other farm animals (e.g., sheep and piglets) using other experimental models, like the flooding dose method and/or the use of radioisotopes, have shown higher tissue protein synthesis rates for the liver, gut, and skeletal muscle tissues [38], [40], [41]. As described by G.E. Lobley [40], the small intestine protein synthesis rates of sheep (a ruminant) has been measured at 45.0%•d^-1^, a value that is ∼7–fold higher than the data we observed in the lactating dairy cow. Baracos *et al.* [17] reported tissue protein synthesis rates values of ∼42.0, 20.0, and 13.0%•d^-1^ in the udder, kidney, and liver, respectively, of a lactating goat. Again, these tissue protein FSR values in the goat are 5.0, 1.7, 2.2-fold higher for the udder, kidney, and liver, respectively, than we have reported in this paper for the cow. Both the prolonged 96 h measurement period [34], especially problematic for the organs with rapid protein export/turnover rates, and the use of the extracellular plasma free enrichment [42] likely contribute to the lower reported tissue protein synthesis rates in the lactating dairy cow. It is often difficult to compare tissue protein FSR values between studies that use vastly different experimental approaches (various mammalian species, precursor pool selection, etc.), but we emphasize that the FSR values should be considered crude (under) estimates. Overall, the presented FSR values provide comparative values, and unique insight, between a large variety of organ and skeletal muscle tissues within a single study and the same experimental approach. Our data highlight that there are high tissue protein synthesis rates in organs of a lactating cow, and these values are much higher than skeletal muscle protein synthesis rates.

In human nutrition research the amino acid composition of a protein-rich food is one of the characteristics used to predict its anabolic potential for the stimulation of postprandial muscle protein synthesis rates [43], and it is often proposed that a high quality protein source has a similar amino acid concentration to that of human body protein [44]. The evidence suggests that milk-derived whey protein is the most anabolic, when compared with other dietary protein sources, allowing for the maximal stimulation of postprandial muscle protein synthesis rates. It is assumed that the high(er) leucine content of whey protein is critically important to maximize postprandial muscle protein synthesis rates [2], [45], [46]. We assessed the protein-bound amino acid concentrations of a piece of the beef (foreshank), and compared it with whey and casein protein fractionated from the collected dairy milk and a series of human skeletal muscle biopsies that we analyzed. As shown in **Table 2**, the amino acid concentrations are similar between human and cow meat, but the leucine concentration in the meat is lower when compared with the leucine content of milk-derived whey protein (∼9 *vs.* ∼14% leucine by content). While some data suggest that beef is an effective food source to stimulate muscle protein synthesis rates [47], it remains to be investigated whether beef ingestion stimulates postprandial muscle protein synthesis rates of equal magnitude when compared with the ingestion of iso-nitrogenous amount of casein or whey protein.

In conclusion, our data shows there is large variance in L-[1-^13^C]phenylalanine enrichments between the various meat cuts, but the enrichment value is quite minimal when comparing the meat enrichments with the various organs. We provide evidence that there are high protein turnover rates in the kidney, udder, and liver of a lactating dairy cow. The infusion of a large amount of L-[1-^13^C]phenylalanine produces ∼210 kg of intrinsically labeled beef for use in the study of protein digestion and amino acid absorption kinetics *in vivo* in humans. The relative proportion of the measured protein-bound essential amino acid concentrations is similar between human skeletal muscle and cow meat. Further work is warranted to assess the effectiveness of beef for the stimulation of postprandial skeletal muscle anabolism when compared against other protein-rich food sources.
